# Distribution of Metals in the Termite *Tumulitermes tumuli* (Froggatt): Two Types of Malpighian Tubule Concretion Host Zn and Ca Mutually Exclusively

**DOI:** 10.1371/journal.pone.0027578

**Published:** 2011-11-08

**Authors:** Aaron D. Stewart, Ravi R. Anand, Jamie S. Laird, Michael Verrall, Chris G. Ryan, Martin D. de Jonge, David Paterson, Daryl L. Howard

**Affiliations:** 1 CSIRO Earth Science and Resource Engineering, Perth, Australia; 2 CSIRO Earth Science and Resource Engineering, School of Physics, University of Melbourne, Melbourne, Australia; 3 University of Tasmania Centre of Excellence in Ore Deposits (CODES), Hobart, Australia; 4 Australian Synchrotron, Clayton, Australia; University of Osnabrueck, Germany

## Abstract

The aim of this study was to determine specific distribution of metals in the termite *Tumulitermes tumuli* (Froggatt) and identify specific organs within the termite that host elevated metals and therefore play an important role in the regulation and transfer of these back into the environment. Like other insects, termites bio-accumulate essential metals to reinforce cuticular structures and utilize storage detoxification for other metals including Ca, P, Mg and K. Previously, Mn and Zn have been found concentrated in mandible tips and are associated with increased hardness whereas Ca, P, Mg and K are accumulated in Malpighian tubules. Using high resolution Particle Induced X-Ray Emission (PIXE) mapping of whole termites and Scanning Electron Microscope (SEM) Energy Dispersive X-ray (EDX) spot analysis, localised accumulations of metals in the termite *T. tumuli* were identified. *Tumulitermes tumuli* was found to have proportionally high Mn concentrations in mandible tips. Malpighian tubules had significant enrichment of Zn (1.6%), Mg (4.9%), P (6.8%), Ca (2.7%) and K (2.4%). Synchrotron scanning X-ray Fluorescence Microprobe (XFM) mapping demonstrated two different concretion types defined by the mutually exclusive presence of Ca and Zn. In-situ SEM EDX realisation of these concretions is problematic due to the excitation volume caused by operating conditions required to detect minor amounts of Zn in the presence of significant amounts of Na. For this reason, previous researchers have not demonstrated this surprising finding.

## Introduction

The aim of this research was to identify specific organs within the termite *Tumulitermes tumuli* (Froggatt) that host metals and other elements at elevated concentrations and therefore play an important role in the regulation and transfer of these back into the environment. Termites bio-accumulate metals within specific organs and in particular Mn and Zn are concentrated in mandible tips and associated with increased hardness [1]. Digestive tracts of termites are also known to harbour proportionally higher concentrations of metals than the rest of the body including Na, Mg, Al, P, Ca, Mn and Zn [2]. Many metals are important biologically as essential elements or as toxicants, consequently insects have the ability to regulate the internal concentrations of these metals e.g. *Poecilus cupreus* L. maintains homeostatic Zn concentrations but not Cd [3].

Termites are significant agents of ecosystem processes. They can have more biomass than mammals on some African savannas and can remove and digest the majority of plant-originated litter [4], [5], [6]. Termites have the ability to burrow to the subsoil and contribute to the development of soil profiles through bioturbation [7], [8], [9]. Consequently, termite nest structures have long been used as geochemical and mineralogical sample media for the discovery of ore deposits buried beneath weathered cover and shallow sediments [10], [11], [12], [13], [14]. Biologically-essential macronutrients acquired through food sources, including Mg, Ca, Zn, P and K, are found in termite nests at concentrations above those in adjacent soils [7], [15], [16], [17]. This occurs to the extent that mounds may be used as mineral licks by animals [18]. The erosion of termite mounds adds elements to nearby soil, creating termite-induced heterogeneity, with the mounds acting as reservoirs [7].

Ingestion of environmental metals, particularly heavy metals, may result in accumulation within individual termites. Storage detoxification, which leads to elevated concentrations of metals in organisms, does not appear to be as pronounced in insects as in other terrestrial invertebrates such as molluscs [19]. However, ants are capable of accumulating metals more effectively than many insects [20], and consequently have been shown to be effective bio-indicators of the concentration of metals in the environment [21], [22], [23]. Metal accumulation in ants is effected by species specific uptake patterns, caste, developmental stage and seasonal variation [20], [24], [25]. The dynamics of metal absorption and elimination vary with different metals, particularly between heavy metals and essential elements. For example, Carabid beetles have been shown to accumulate Cd until reaching a plateau in concentration. Upon withdrawal from exposure Cd is then rapidly eliminated [3], [26]. Elimination may also happen at the time of metamorphosis [27]. However, Zn concentrations are maintained consistently even with elevated exposure in diet [3].

Termites with centralised nest structures differ from many insects in that their waste products are locally concentrated to their nest. This gives rise to the possibility that termite mounds may harbour elevated metal concentrations as a result of excretion, even if individual termites do not absorb metals substantially or at all during feeding.

Investigations utilizing Particle Induced X-ray Emission (PIXE) analysis have taken advantage of its capacity to make quantifiable analysis on small samples, usually by homogenising samples of insect parts e.g. termite [2], whole insect, e.g. mosquito [28] and beetle [29]. PIXE has been used to map specific tissues within insects including the mandibles of termites and ants [30], [31], Malpighian tubules of a beetle [32], grasshopper brain [33], [34] and silk moth silk glands [35]. The penetrative power of the PIXE analysis has allowed the mapping of whole insect including ants [30], vinegar fly [36], bugs [37] and housefly [38]. Combined, these characteristics lend PIXE analysis to investigations of insects where identification of tissues, important to metal storage and detoxification within the insect, is required. The main advantages in using PIXE over competing techniques like SEM Energy Dispersive X-ray (EDX) include a higher sensitivity and its non-destructive probing of complete insect structures. The ability to probe without sectioning reduces possible contamination from cutting utensils and importantly, maintains structural integrity i.e. negligible displacement of organs from original locations. The greater spatial resolution of EDX means initial PIXE imaging to map trace element hot-spots can be subsequently followed up by pin-point EDX imaging. Synchrotron X-ray Florescence Microscopy (XFM) is a powerful tool to investigate spatial resolution and to detect low concentrations.

There is no general pathway of metal accumulation apparent in insects studied to date [39]. Evidence is lacking to correlate storage patterns to habitat, diet or insect phylogeny. Organ storage of mineral salts appears to be specific to insect species. For this reason insect species need to be investigated separately. Malpighian tubule mineral concretions with metal incorporation (Zn, Mn and Mg) have previously been described in Malpighian tubules of various insects including the Orthoptera, Coleoptera, Diptera, Lepidoptera, Blattodea, Phasmatodea and Hymenoptera [32], [38], [39], [40].

The exact location of accumulated metals within a termite is investigated here using PIXE imaging, SEM-EDX analysis and Synchrotron XFM.

## Results

**Figure 1:**
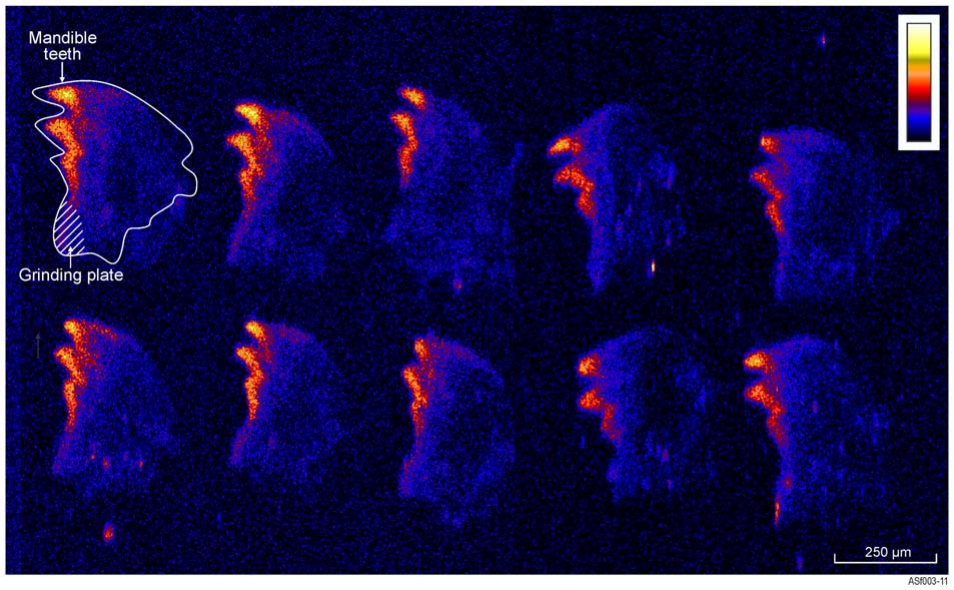
Ten termite mandibles displaying Mn concentrated along the cutting edge. More intense colour indicates higher concentration (Maximum of 2–3% as measured by SEM EDX). Mandibles are orientated with the teeth projecting towards the left of image.

PIXE scans of multiple termite mandibles and SEM/EDX analysis revealed consistent occurrence of Mn along the cutting edge (Figure 1; Table 1). Manganese concentration in termite mandibles as measured by SEM/EDX had a mean concentration of 0.49% (n = 50, SE = 0.051, min = 0.19, max = 1.45 median = 0.34).

**Table 1:** Elemental content of *Tumulitermes tumuli* Malpighian tubule (SEM EDX), soil, nest material and food source (ICP-MS/OES) in ppm (mean (SE)).

Element	Malpighian Tubule	Stored Food	Soil	Nest Material
Al	1797 (178)^a^	16730 (1412)^c^	76994 (2353)^b^	67500 (2077)^b, c^
Na	18629 (1010)^a^	390 (62)^b^	959 (51)^b^	1072 (142)^b^
Mg	49813 (3536)^a^	700 (42)^b^	1172 (67)^b^	1272 (89)^b^
P	68087 (3502)^a^	380 (17)^b^	359 (15)^b^	365 (11)^b^
S	1045 (174)^a^	824 (36)^a^	101 (6)^b^	333 (37)^a,b^
K	23790 (1379)^a^	2505 (130)^b^	8666 (115)^c^	6966 (127)^b,c^
Ca	27306 (3254)^a^	14920 (741)^a,c^	584 (46)^b^	2956 (227)^b,c^
Mn	1148 (124)^a^	262 (17)^b^	422 (35)^b,c^	592 (40)^a,c^
Fe	1116 (134)^a^	12715 (1183)^b^	58919 (600)^b,c^	71838 (2218)^c^
Zn	16035 (1071)^a^	51 (3)^b^	59 (3)^b^	59 (2)^b^

Different letters in the same row indicates significant difference (Dunn's pairwise multiple comparison P<0.05). All metals vary significantly across sample media (ranked ANOVA p<0.001).

**Figure 2:**
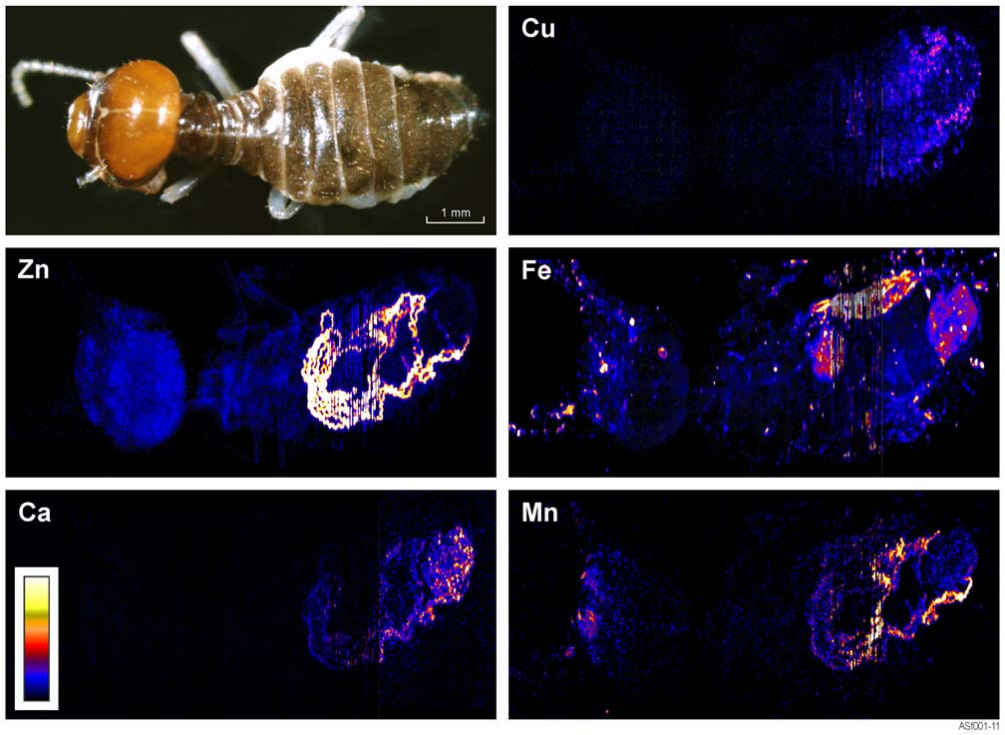
Composite PIXE image of a worker termite (*Tumulitermes tumuli*) showing distribution of 5 metals. More intense colours indicate higher concentrations of metals. Iron appears concentrated around the antennae and lower abdomen; Zn and Ca in the Malpighian tubules with a weak accumulation over the entire body of Zn and Mn within Malpighian tubules and mandibles.

Whole body PIXE imaging revealed locally concentrated levels of Fe, Zn and Mn. Manganese has a mean concentration in the mandible tips of 0.5% (Figure 2). Iron is found throughout the termite with localized spots of higher concentration occurring predominantly in the antennae, head and lower abdominal region. The broad homogenous Fe patch in the rear abdominal region corresponds roughly with the terminal rectum. Higher Zn concentrations appear as lines through the abdominal region corresponding to Malpighian tubules. The presence of zinc in elevated concentrations (≈ 2% as measured with SEM EDX) was predominantly evident in abdominal structures (Malpighian tubules).

**Figure 3:**
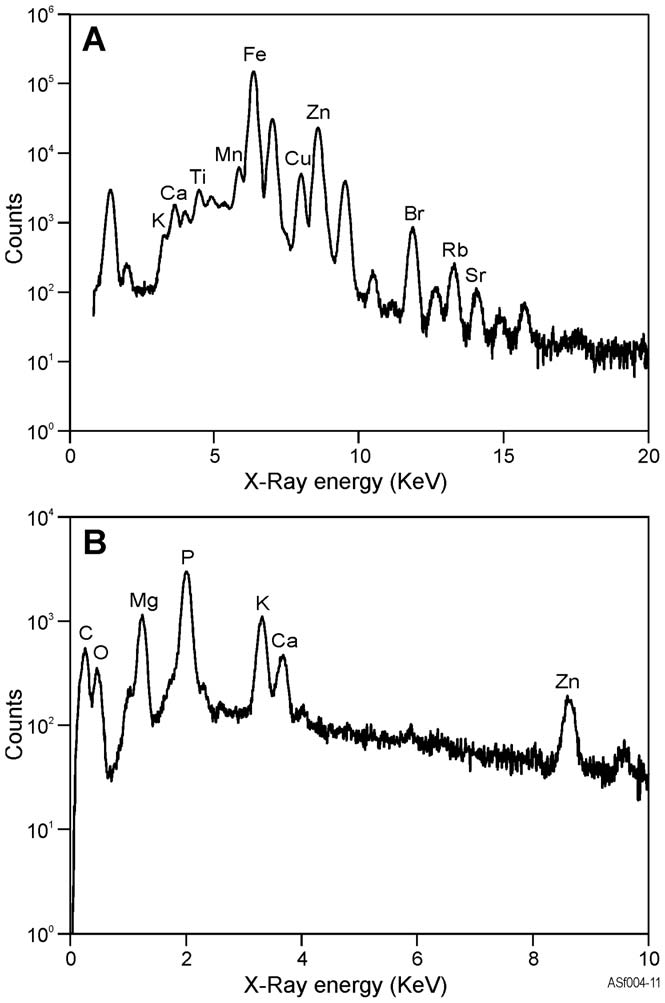
Excited X-ray spectra. A: Whole termite body PIXE scan. B: SEM scan of the Malpighian tubule.

In addition to Fe, Zn and Mn, the elements Ca, Cu and Br are at levels high enough in the abdomen for PIXE mapping (Figure 2). Zinc, Cu, Mn and Br appear confined to specific anatomical structures, predominantly the Malpighian tubules, however Ca and Fe have a much more scattered distribution. Some Fe recorded in the analyses may be a result of contamination from Fe-rich soil which the termite was collected from. Concretions within the Malpighian tubule were confirmed as the host of high concentrations of Zn via SEM and EDX analysis. The concretions are located within the cells of the Malpighian tubule and vary in size from 1–5 µm. Figure 3 presents EDX spectra for the Malpighian tubule SEM scan and the whole body PIXE scan corresponding EDX spectrum.

**Figure 4:**
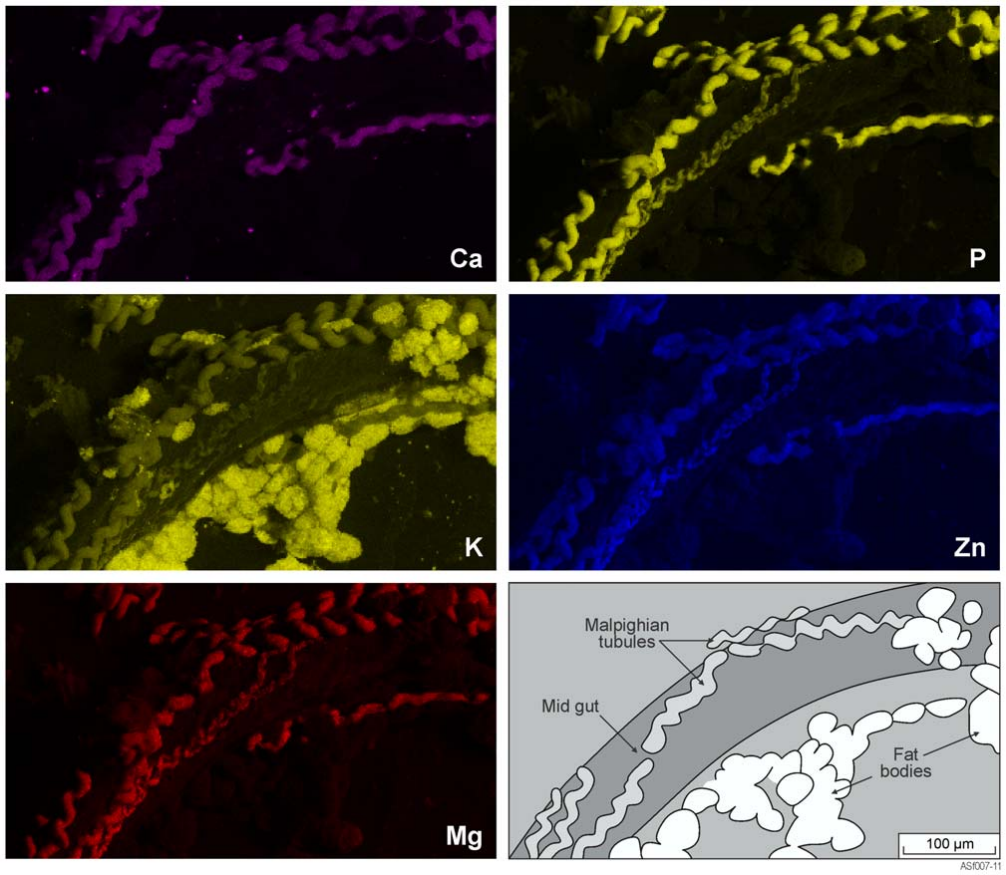
EDX maps of Malpighian tubules in association with the mid gut. More intense colour indicates higher concentrations. Calcium, Mg, P and Zn are delineated within Malpighian tubules. Potassium is associated with fat bodies lying adjacent to the Malpighian tubules.

EDX extracted elemental concentrations from the same tubule, indicated accumulation of several metals including Zn (Table 1). The accumulation of the higher concentration metals can be clearly seen with EDX elemental mapping (Figure 4). The underlying mid gut is lower in concentrations of Ca, K, Mg, P and Zn compared with Malpighian tubules. SEM EDX is not sensitive enough to image Mn effectively. Potassium is also found in fat bodies adjacent to the Malpighian tubules. Aluminium, K, and Fe all occur in soil at higher concentrations than in the food source (∼ 4–5 fold).

**Figure 5:**
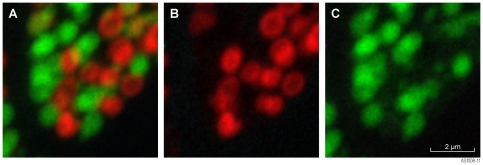
Synchrotron XFM images of Malpighian tubule concretions. Scan size 8.0×8.0 µm^2^, 80×80 pixels, 1 s dwell per pixel. A: Composite image map of Zn and Ca (red and green). B: Zn map (red), C: Ca map (green). Image A demonstrates that high concentrations of Zn and Ca occur in mutually exclusive concretions.

Synchrotron XFM imaging of sectioned Malpighian tubule concretions revealed detectable quantities of Mn, Zn, Ca, and P within the spherical concretions. Phosphorous and Mn were present in all imaged concretions at roughly the same relative concentration. However concretions display a mutually exclusive occurrence of Zn and Ca. There was no Zn detectable in concretions with Ca and vice versa (Figure 5).

## Discussion

The use of Synchrotron XRF (SXRF) provided resolution capable of differentiation of concretion types and the relative composition of individual concretions. SXRF imaging of termite excretory system using a silicon-drift X-ray detector demonstrated 2 types of mineralised concretions adjacent to each other (<1 µm) within the excretory system of the termite. In-situ SEM EDX realisation of these concretions is problematic due to the excitation volume caused by operating conditions required to detect minor amounts of Zn in the presence of significant amounts of Na. For this reason previous researchers have not demonstrated this surprising finding. Like the study here, Tylko [38] found Ca and Zn to accumulate in association within Malpighian tubules of the housefly *Musca domestica* L. However, specific location within the tubules of Zn and Ca may well be isolated to separate concretions as discovered here. Generally, the composition of Ca-containing granules in invertebrates is quite variable [41]. Here evidence of the high concentration of P in SEM EDX lends support to the occurrence of Ca as a phosphate, perhaps as apatite. Phosphorous was present at equal concentration in Ca and Zn bearing concretions. Brown's [41] review suggests that there is a general trend for occurrence of Zn in the same bodies as Ca. However, techniques used need to ensure that individual concretions are being targeted for analysis. As shown here concretions with markedly different composition can occur in close proximity. Waterhouse [42] noted at least 8 morphologically distinct concretion-like structures within the tubules, some of these morphological variations may represent concretions at different stages of formation and or excretion or unrelated structures. Several “types” of concretion have been described by various authors, including Sohal [43] who described 1 luminal and 3 intra cellular types of concretions (a, b and c) from Malpighian tubules of the housefly *Musca domestica* using TEM EDX and WDX. Interestingly, Sohal [43] found that across the 4 types of concretions Zn concentration has an inverse relationship with Ca. Wessing [44] described 2 types of concretion in *Drosophila* Malpighian tubules. Type 1 being principally consisting of Mg, P and Ca to a much lesser extent K, and type 2 consisting of K and to a lesser extent Mg. Our results are consistent with Wessing's [44] occurrence of type 1 and 2 concretions within the termite although XFM suggested similar levels of P across all concretions. Due to the volume of Malpighian tubules relative to the rest of the insect and the relative concentration of Ca in Malpighian tubules, the majority of Ca within the Drosophila occurs within the tubules [40]. This is likely the case for Ca and Zn in the termite studied here. The implication of this is that concretions represent the principal form in which these metals are retuned to the soil from termites either as excretion or after termite mortality. Zinc containing concretions shown here are likely to be present as zinc phosphate, bound to metallothioneins, or other sulphur bearing amino acids cysteine, cystine or methionine [16]. Calcium, Mg, K, are more likely to be present as phosphates [14], although Maddrell [20] provided evidence that Ca is deposited in an amorphous form in Malpighian tubules of the blood feeding bug *Rhodnius prolixus* Stal.

The distribution of metals within *T. tumuli* suggests a process of absorption through the gut into the haemolymph and subsequent detoxification though Malpighian tubule concretions. These observations are similar to those of Yoshimura [2] who noted higher total concentrations of metals from dissections of the gut region than other parts of the termite *Coptotermes formosanus* Shiraki. This was particularly true for Mg, Al, P, Ca, Zn. Manganese and Cu were found in higher concentrations in the mandible in accord with the findings of Cribb et al. [45] for *C. formosanus*, indicating a mineralisation pattern similar to the termite examined here. Our results demonstrate that it is the Malpighian tubules in close association with the gut that harbor these metals. This is likely to be the case for *C. formosanus* as it is for *T. tumuli*. As an example of how metal accumulation can differ between insects, the ant *Tapinoma sessile* (Say) stores Zn throughout its body and has enriched mandibles [30], which is a common generalised storage pattern for ants where accumulation pattern includes Malpighian tubules and midgut [22].

There is a general trend for the composition of insect diet to influence Malpighian tubule concretion composition [39], [42]. For example, feeding salts of Mg and Ca increases size and number of concretions in the blowfly *Lucilia cuprina* (Wiedemann) larvae [42]. Consequently, it may be important to consider the number and size of concretions and not just specific concentration makeup when making assessments of environment induced variation within insects. With the termite *Reticulitermes flavipes* Kollar changes in food composition had little effect on concentrations in workers for most metals examined. However, worker termites fed an enriched diet significantly increased Ca but decreased Zn. The reduction in Zn concentration observed by Judd and Fasnacht [46] may be a result of hyper compensation. Some interpretation difficulty remains as it is not clear what period of time is required for uptake or the role of periodic changes around ecdysis. There are processes evident that serve to maintain the whole organism Zn and other metal concentrations at relatively stable and food/substrate independent concentrations. Kramarz [3] found the concentration of Zn constant in whole animal analysis of the Carabid beetle *Poecilus cupreus* L. despite changes in diet. However, the mutually exclusive nature of Ca and Zn within different concretions necessitates the consideration of separate excretory processes. After absorption into the haemolymph, maintenance of body concentrations of Zn requires precipitation within concretions of the Malpighian tubules. It is unlikely that the concentration within concretions or the number of concretions within Malpighian tubules can adequately explain the maintenance of whole body concentration over the lifetime of the insect due to there relative volume. Therefore expulsion of concretions is likely to increase in response to substrate/food concentration increases. In this model, concretions in Malpighian tubules act as a form of regulated storage as suggested by Schofield et al. [36]. Excretion of concretions from the Malpighian tubules would explain the ability of termites to hyper-compensate environmental levels of Zn.

PIXE maps revealed that some portions of Malpighian tubule are rich in Zn and less so in Mn and vice versa. This is consistent with Schofield et al. [36] who found non homogenous distribution of Zn throughout the Malpighian tubules of *Drosophila*. The specific location within the Malpighian tubules of Zn enrichment may affect the ability to excrete into the alimentary canal with proximal sections more easily voided. Future research needs to be conducted to determine if substrate or food metal concentration variations are reflected in concretions and the nature and magnitude of the mineralisation within the excretory structures.

## Materials and Methods

### Termite collection

*Tumulitermes tumuli* termites were collected from 10 epigeal (above ground nests to 60 cm in height) nests near the Bentley Volcanic Massive Sulphide deposit 305 km north of Kalgoorlie Western Australia in an area dominated by mulga (*Acacia aneura* Muell. ex Benth). Termites were separated from nest material and placed immediately into 95% ethanol. No specific permits were required for the described field studies. No specific permissions were required for collection of insects form this location. Field studies did not involve endangered or protected species and were conducted on mining leasehold land (Jabiru Metals). Access permission was granted by Jabiru Metals.

### *SEM EDX analysis*

Five termites from each of the 10 nests were dissected and mounted on carbon stubs and carbon coated for examination in a Philips (FEI) XL40 controlled pressure Scanning Electron Microscope (SEM) fitted with EDX. The SEM was operated in high vacuum mode and images were collected with a Robinson backscattered electron detector (BSE). EDX analysis specifically targeted the proximal 1/3 of the Malpighian tubules. One analysis was performed on malpighian tubules for each of 50 termites. One analysis of the apical tip of one mandible from each of 50 termites was also taken. The SEM was set to 8 mm working distance with a 30 kV beam recording 30 seconds live time. No suitable standard is available to match material being assessed, therefore standardless quantification was used with ZAF matrix correction. It should be noted that the X-ray excitation volume was expected to exceed the volume of individual concretions within the Malpighian tubule due to the high carbon content of the organic material. Results therefore represent an averaging of the local environment using several concretions and Malpighian tubule epithelial tissues. An EDX map of one dissected mid gut area was also performed.

### PIXE analysis

PIXE imaging of one termite mandible from each nest was conducted. PIXE analysis of mandibles and SEM EDX of concretions found high concentrations of Mn within the mandible and Zn within the Malpighian tubule consistently across individual termites and across nests. For this reason one termite was selected for full body PIXE analysis.

For PIXE imaging, a single termite (Figure 2) was pressed between two glass slides to approximately 200 µm thickness, air dried and mounted on a carbon stub. The CSIRO nuclear microprobe beamline provided a beam of 3.0 MeV protons focused to a ∼2 µm spot [47]. Scanning in X-step mode (a combination of electrostatic scanning in Y and stage scanning in X) made a series of two scans covering the near entirety of a single termite (2 mm×6 mm). The deep penetration depth of the proton beam eliminated the need for sectioning of the sample, providing good elemental contrast of internal organs. Mapping and quantification was conducted with the GeoPIXE software [48], [49], [50].

### Food, nest material and soil analysis

**Figure 6:**
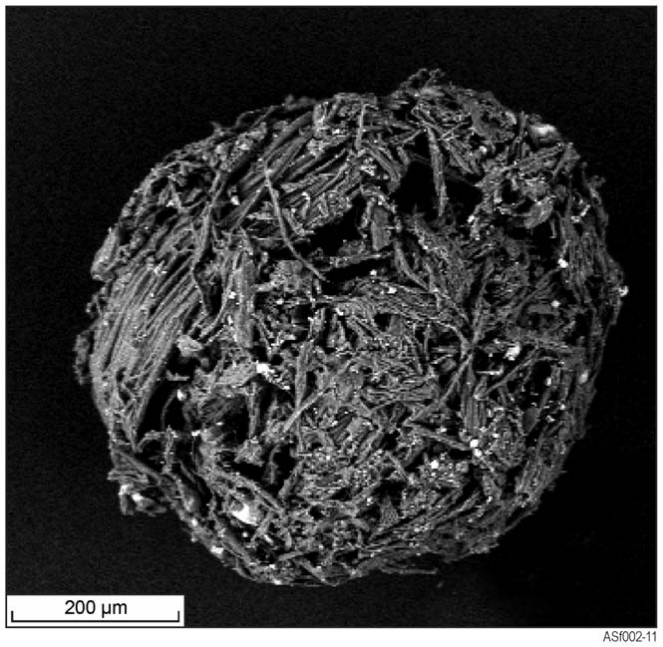
SEM image of a stored food sphere. Sphere consists of masticated grass, mulga leaves and other surface material.

*Tumulitermes tumuli* feeds on surface litter including mulga leaves and grasses which is stored in the form of small 500 µm diameter masticated spheres (Figure 6). Large quantities of this material are typically found in the upper portion of the epigeal nest. From each nest a sample of stored food was taken for analysis. Additionally, inner nest material and a soil sample (2 m from the nest at 10–20 cm depth) were also collected for comparative analysis. Soil, nest carton (sieved to <250 µm) and stored food was analysed via aqua regia Inductively Coupled Plasma - Mass Spectrometry/Optical Emission Spectrometry (ICP-MS/OES) at Ultratrace Laboratories, Perth.

### Synchrotron XFM imaging

For Synchrotron XFM imaging, whole Malpighian tubules were dissected and set in Spurr's resin and cured at 70°C for 12 hours before sectioning to 300 nm with a Leica EM UC6 microtome at the Centre for Microscopy, Characterisation and Analysis (CMCA), University of Western Australia. No tissue fixation was performed.

SXRF images were acquired using the zone-plate nanoprobe instrument of the XFM beamline at the Australian Synchrotron [51]. A 9.76 keV photon beam was focussed into a 100 nm beam-spot, through which the sample was raster scanned in 100 nm steps over an area of 8.0×8.0 µm. At each point in the raster fluorescence spectra were acquired for 1 second using a single element silicon-drift X-ray detector (SII Vortex) which was oriented at 107 degrees to the beam axis.
